# Supplementary material for: Dynamic actuation enhances transport and extends therapeutic lifespan in an implantable drug delivery platform
Source: Nat Commun. 2022 Aug 3;13:4496. doi: 10.1038/s41467-022-32147-w (PMC9349266; doi:10.1038/s41467-022-32147-w)
Supplement: Supplementary file 1 — Supplementary Information [file 41467_2022_32147_MOESM1_ESM.pdf]

Dynamic actuation enhances transport and extends therapeutic lifespan in an implantable drug delivery platform

William Whyte [1,†], Debkalpa Goswami [1,†], Sophie X. Wang [1,2,†], Yiling Fan [3], Niamh A. Ward [1,4], Ruth E. Levey [5], Rachel Beatty [5], Scott T. Robinson [5,6], Declan Sheppard [7], Raymond O'Connor [5], David S. Monahan [1,5], Lesley Trask [4], Keegan L. Mendez [8], Claudia E. Varela [8], Markus A. Horvath [8], Robert Wylie [5], Joanne O'Dwyer [1,4], Daniel A. Domingo-Lopez [5], Arielle S. Rothman [1], Garry P. Duffy [5,6], Eimear B. Dolan [4,*], Ellen T. Roche [1,3,8,*]

[1] Institute for Medical Engineering and Science, Massachusetts Institute of Technology, Cambridge, MA, USA.

[2] Department of Surgery, Beth Israel Deaconess Medical Center, Boston, MA, USA.

[3] Department of Mechanical Engineering, Massachusetts Institute of Technology, Cambridge, MA, USA.

[4] Department of Biomedical Engineering, National University of Ireland Galway, Galway, Ireland.

[5] Anatomy and Regenerative Medicine Institute (REMEDI), National University of Ireland Galway, Galway, Ireland.

[6] Advanced Materials and BioEngineering Research Centre (AMBER), Trinity College Dublin, Dublin, Ireland.

[7] Department of Radiology, University Hospital, Galway, Ireland.

[8] Harvard-MIT Program in Health Sciences and Technology, Cambridge, MA, USA.

[†] These authors contributed equally: William Whyte, Debkalpa Goswami, Sophie X. Wang

[*] Corresponding author email: eimear.dolan@nuigalway.ie; etr@mit.edu

Supplementary Figures

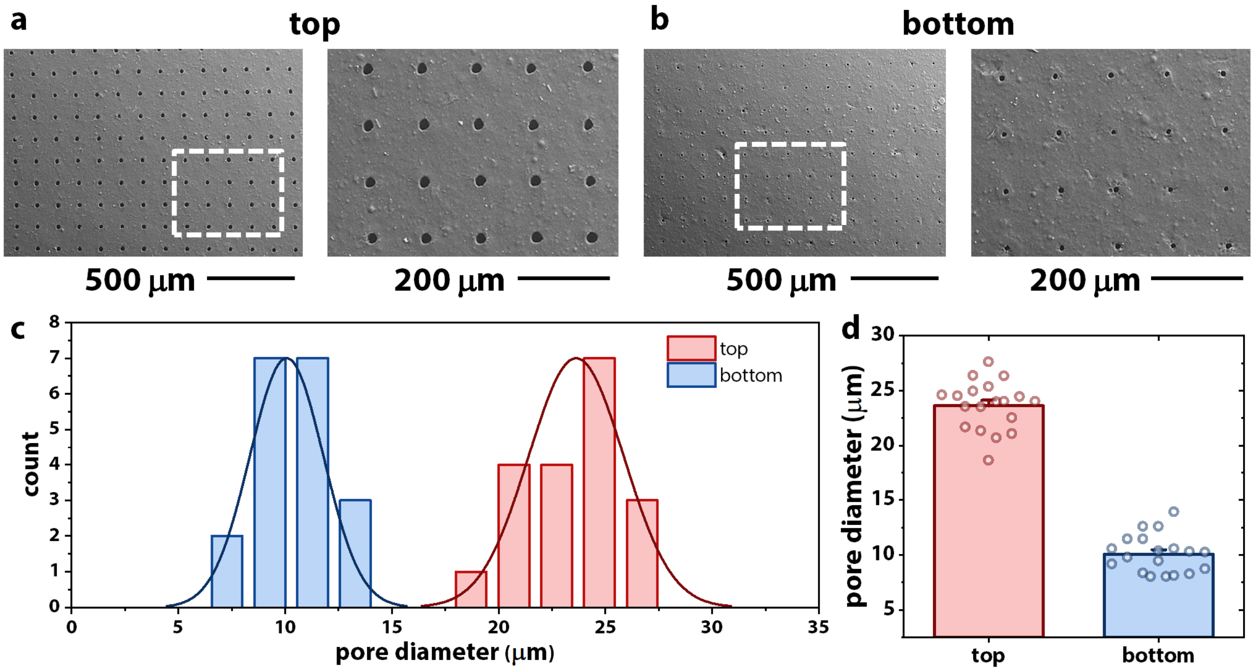

Supplementary Fig. 1 | STAR porous membrane characterization. Scanning electron micrographs of the laser cut TPU membrane from the **a**, top and **b**, bottom view. **c**, Pore size distribution at the top and bottom of the membrane. **d**, Average pore diameter at the top and bottom of the membrane. $n = 19$ pores from single micrograph. Data are means ± standard error of mean.

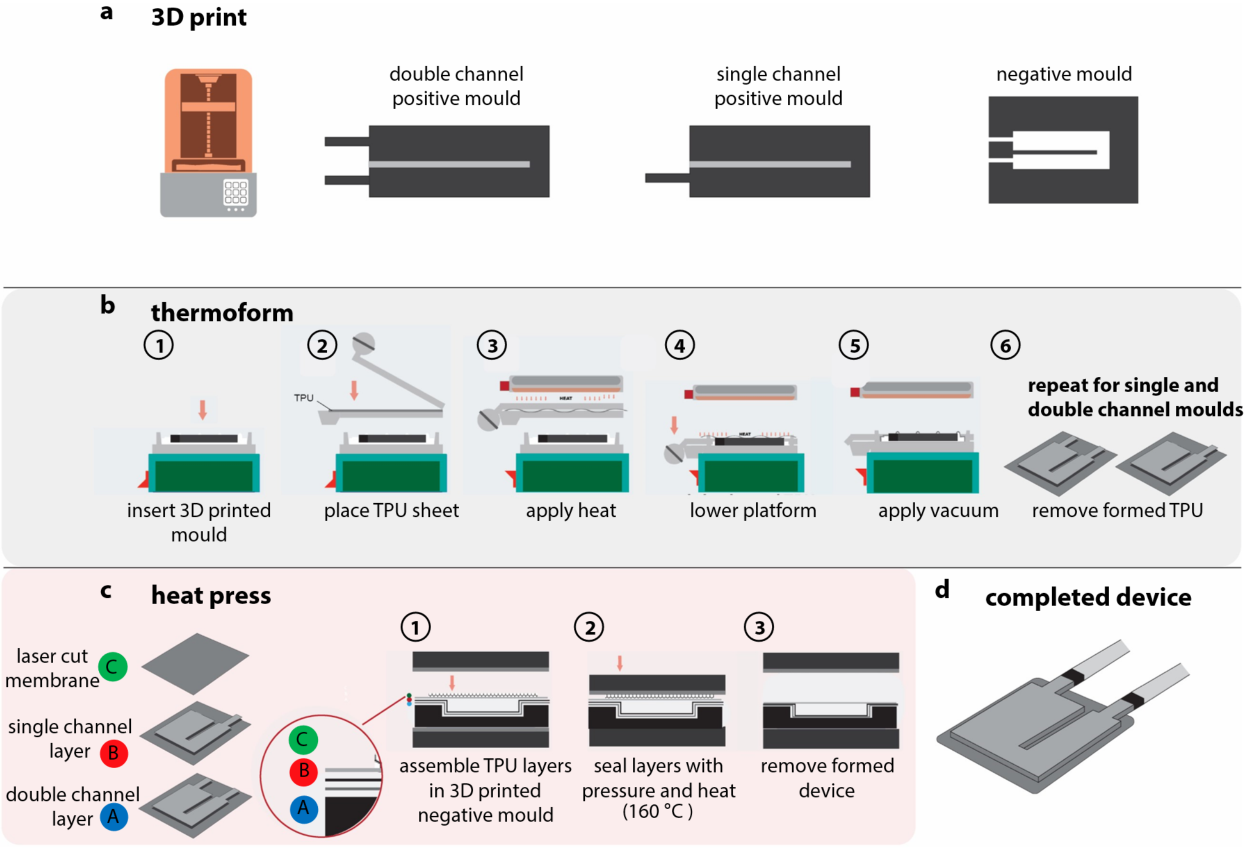

Supplementary Fig. 2 | STAR Manufacturing Process. a, 3D printing of positive and negative moulds. **b**, Thermoforming of double and single channel layers using printed positive moulds. **c**, Heat sealing of assembly consisting of double channel layer, single channel layer, and laser cut porous membrane. **d**, Completed STAR device.

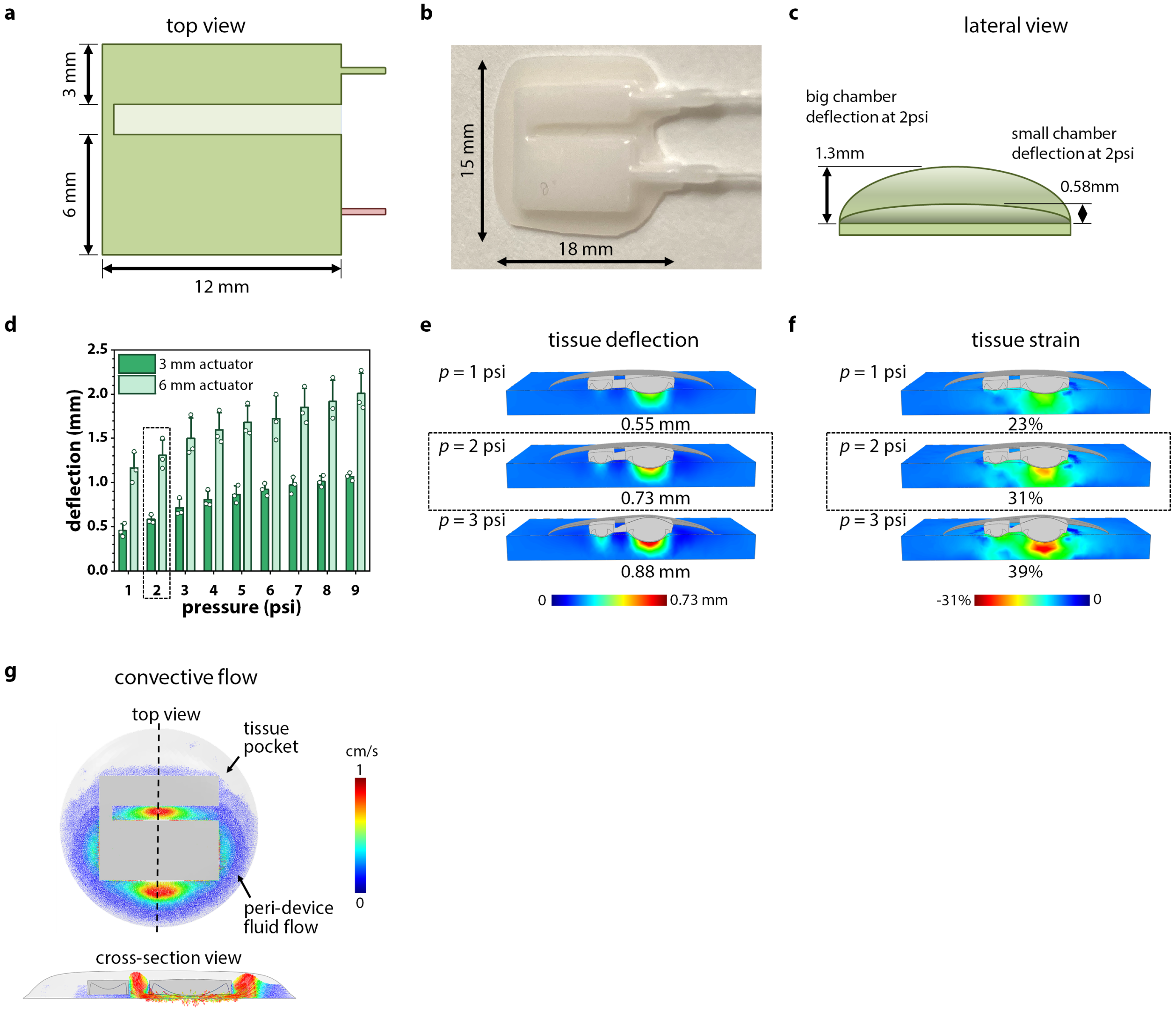

Supplementary Fig. 3 | Membrane deflection in STAR. a, Schematics of bi-chambered STAR device. Single pressure input allows for variable deflection in a single device. **b,** Prototype of bi-chambered STAR device manufactured by the process described in Supplementary Figure 2. **c**, Schematic depicting lateral view of bi-chambered device, and differential deflection height of each chamber upon uniform pressurization. **d,** Maximum membrane deflection for the 3 mm and 6 mm chambers during actuation with a 1–9 psi pressure input. Pressure of 2 psi was chosen for the preclinical experiments. $n = 3$ bi-chambered devices at each actuation pressure. Data are means ± standard error of mean. **e**, Finite element (FE) model showing tissue deflection caused by STAR membrane at 1, 2, and 3 psi pressure input. **f**, FE model showing tissue strain caused by STAR membrane at 1, 2, and 3 psi pressure input. **g**, FE model showing peri-implant fluid velocity of convective flow around the large and small chamber during actuation at 2 psi.

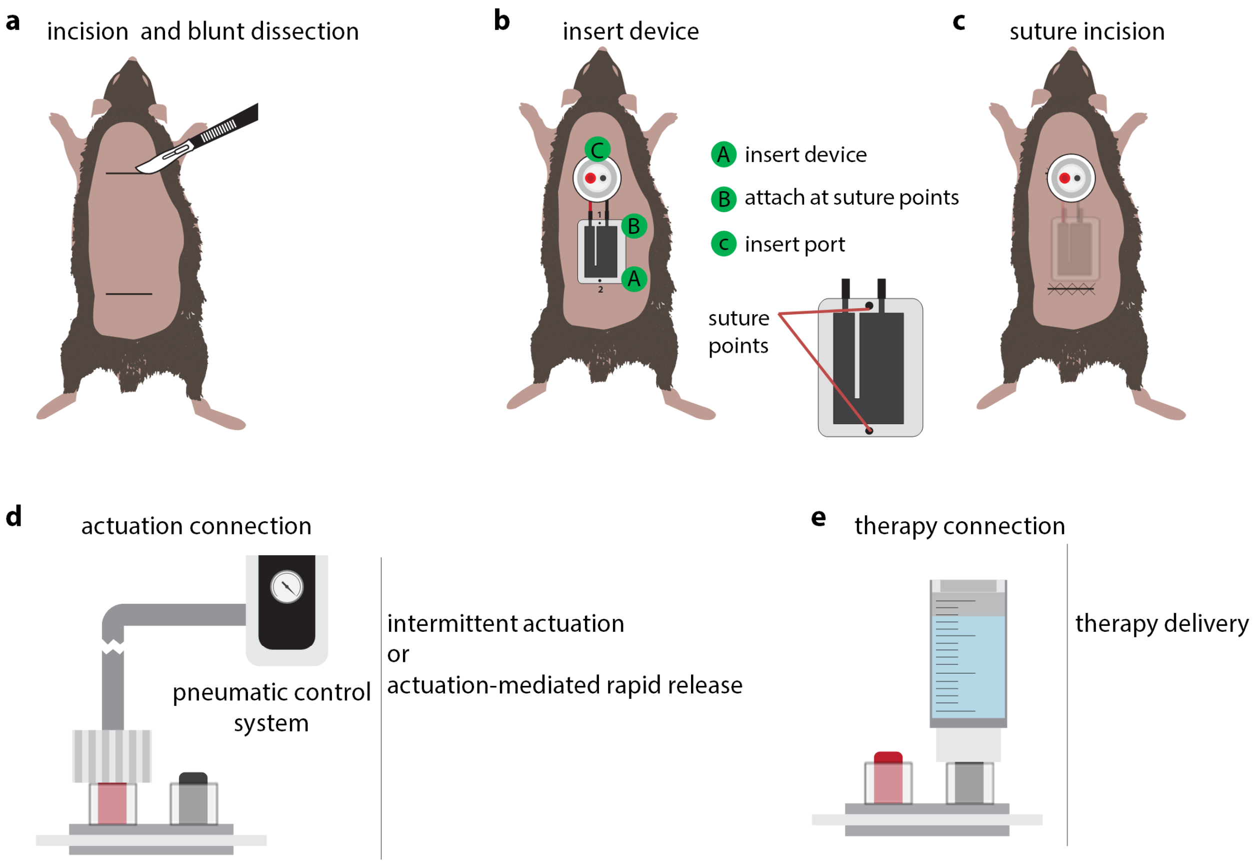

Supplementary Fig. 4 | Overview of the STAR small animal surgical model. a, Incision and blunt dissection. **b**, Insertion of the device, port, and attachment to the underlying fascia at designated suture points. **c**, Closing of the skin surgical incision with sutures. **d**, Connection of pneumatic control system to actuation port for delivery of intermittent actuation or actuation-mediated rapid release. **e**, Connection of drug filled syringe to therapy port for replenishable, targeted delivery to the STAR therapy reservoir.

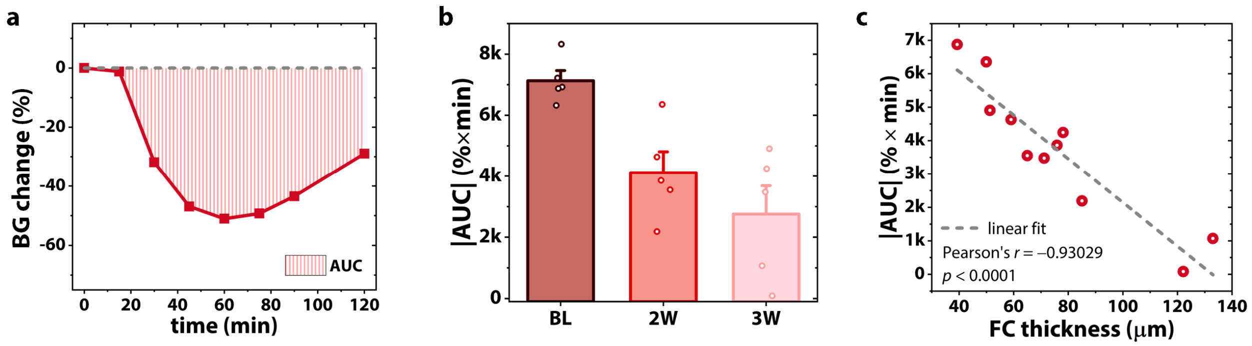

Supplementary Fig. 5 | a, Area under the curve (AUC) calculated by integrating the area under the blood glucose (BG) % curve. **b,** Temporal evolution of AUC, calculated at baseline (BL; 3 days), 2 weeks (2W) and 8 weeks (8W). AUC decreases over time as the fibrous capsule (FC) grows. $n = 5$ mice per timepoint. Data are means ± standard error of mean. **c,** Relationship between FC thickness and AUC. Each data point is a measurement from a different mouse. *p*-value calculated from two-tailed *t*-test of significance of the correlation coefficient.

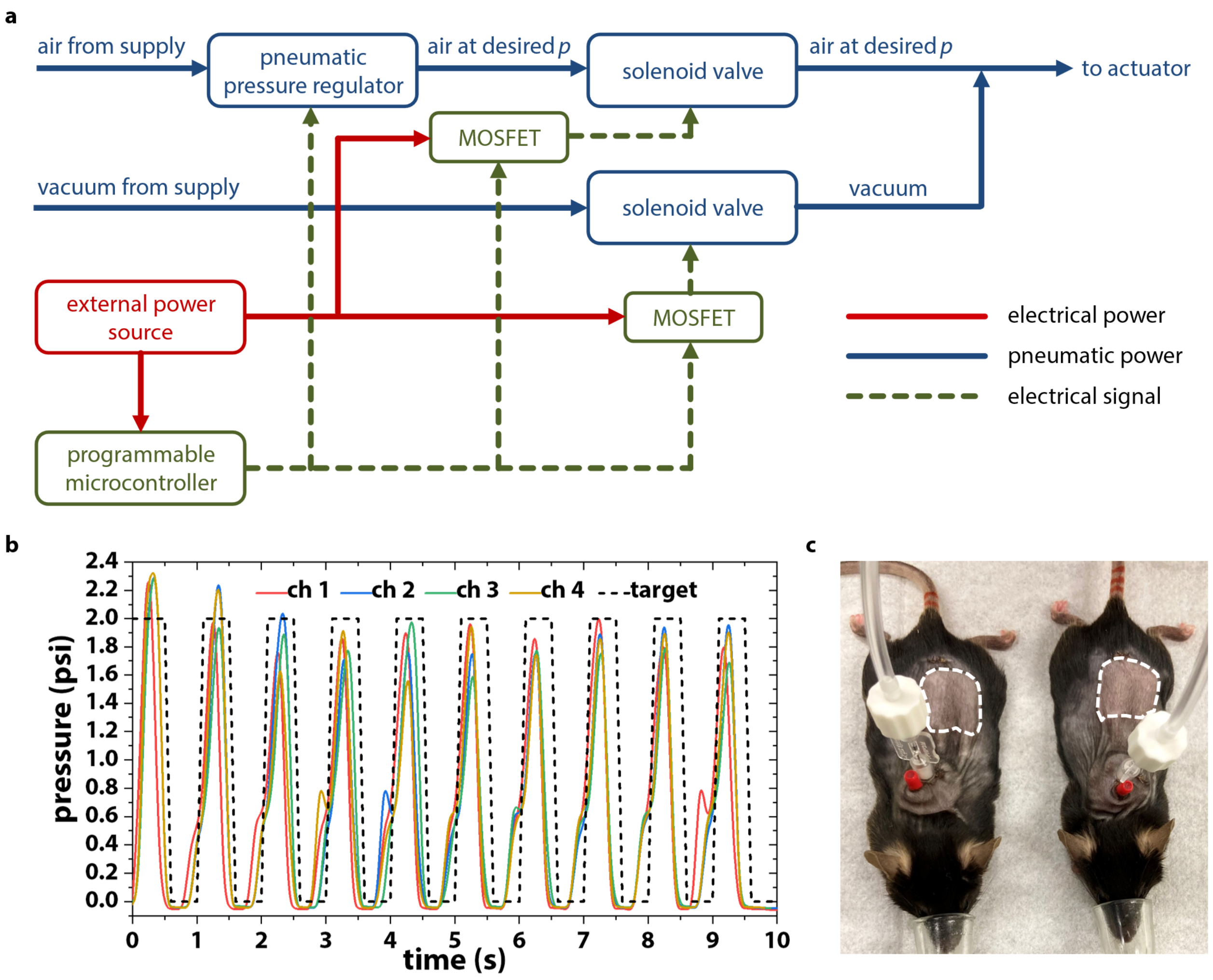

Supplementary Fig. 6 | Development of an electropneumatic control system to actuate STAR. **a**, Block diagram showing the components of the programmable electropneumatic control system used to actuate STAR. **b**, Actuation pressure profile measured by an in-line pressure sensor. **c**, Application of a dynamic actuation regimen *in vivo*.

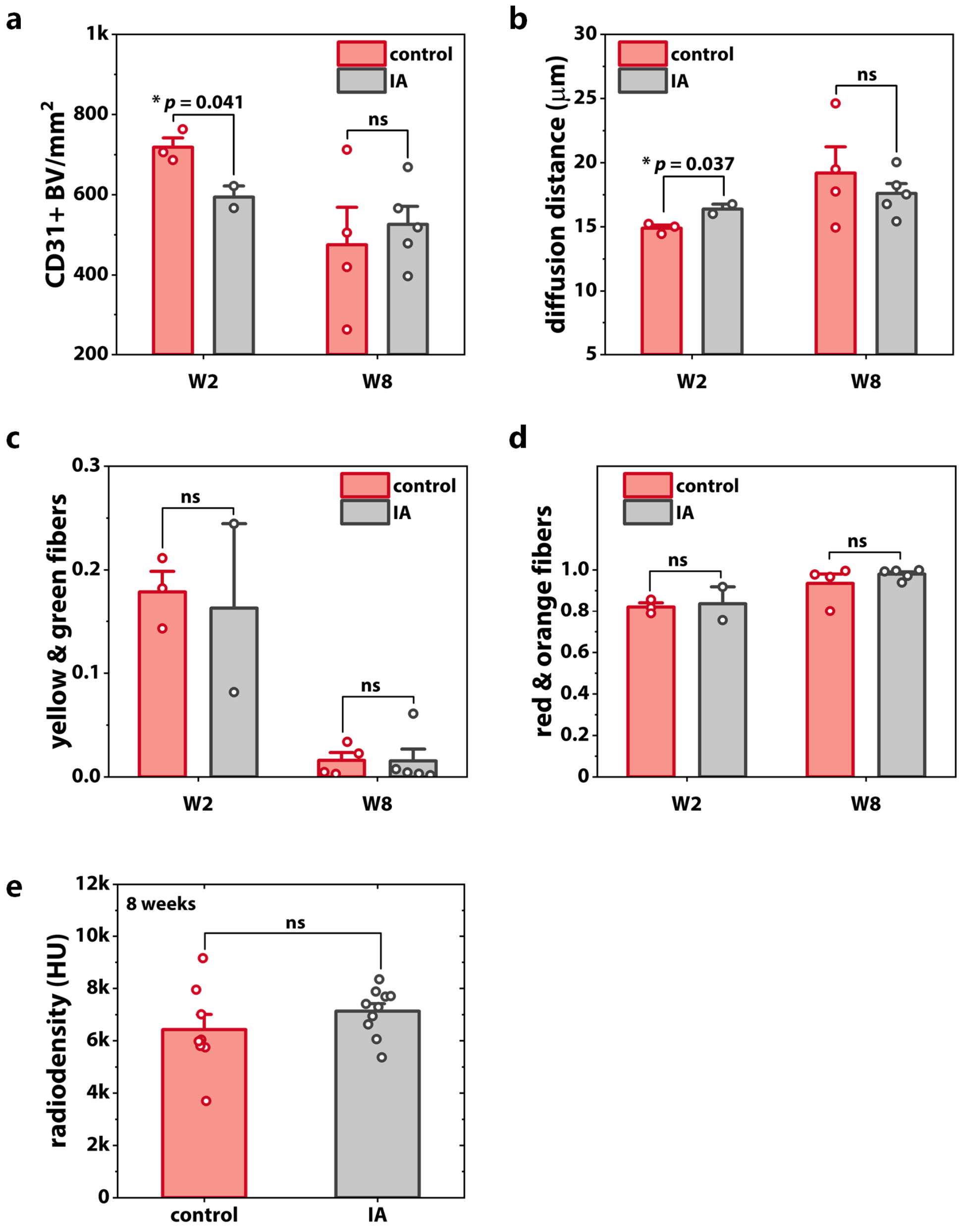

Supplementary Fig. 7 | a, Number of CD31+ blood vessels per mm^2 in control and IA groups at 2 and 8 weeks. **b**, Radial diffusion distance in control and IA groups at 2 and 8 weeks. **c**, **d,** Relative integrated density of immature (**c**) and mature (**d**) collagen fibers in capsule obtained using polarized light microscopy after picrosirius red staining. Orange/red = mature collagen, yellow/green = immature collagen. **e**, Fibrous capsule density expressed as radiodensity (Hounsfield units) in control and IA groups at 2 and 8 weeks. Data are means ± standard error of mean. $n = 2$–5 mice per group. No technical replicates in **a**–**d**; 2 technical replicates per mouse (one per chamber) in **e**. p-values are calculated from unpaired, two-tailed, two-sample t-tests. See Supplementary Note 1 for detailed statistical analyses.

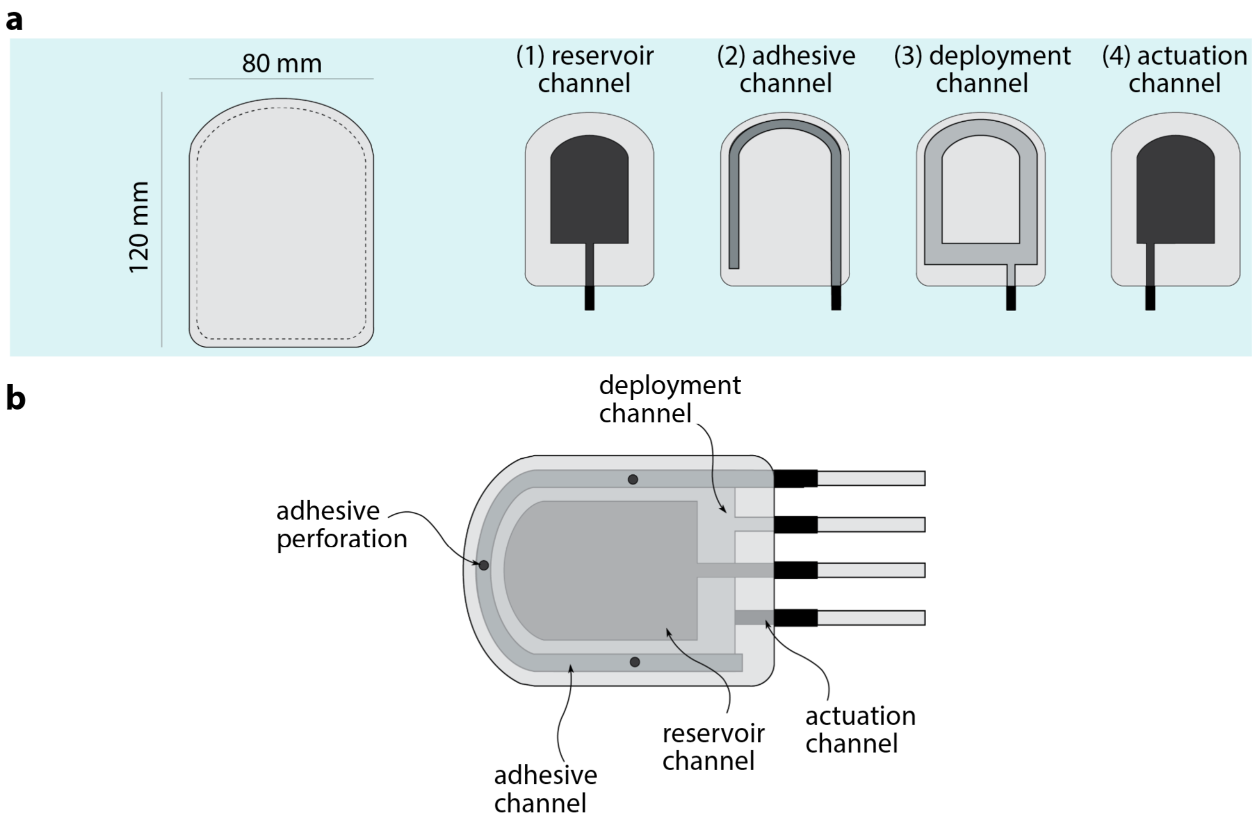

Supplementary Fig. 8 | Schematic of device for human cadaveric model. a, Schematics showing the layers of the scaled-up STAR device designed for minimally invasive clinical translation. **b**, Assembled device.

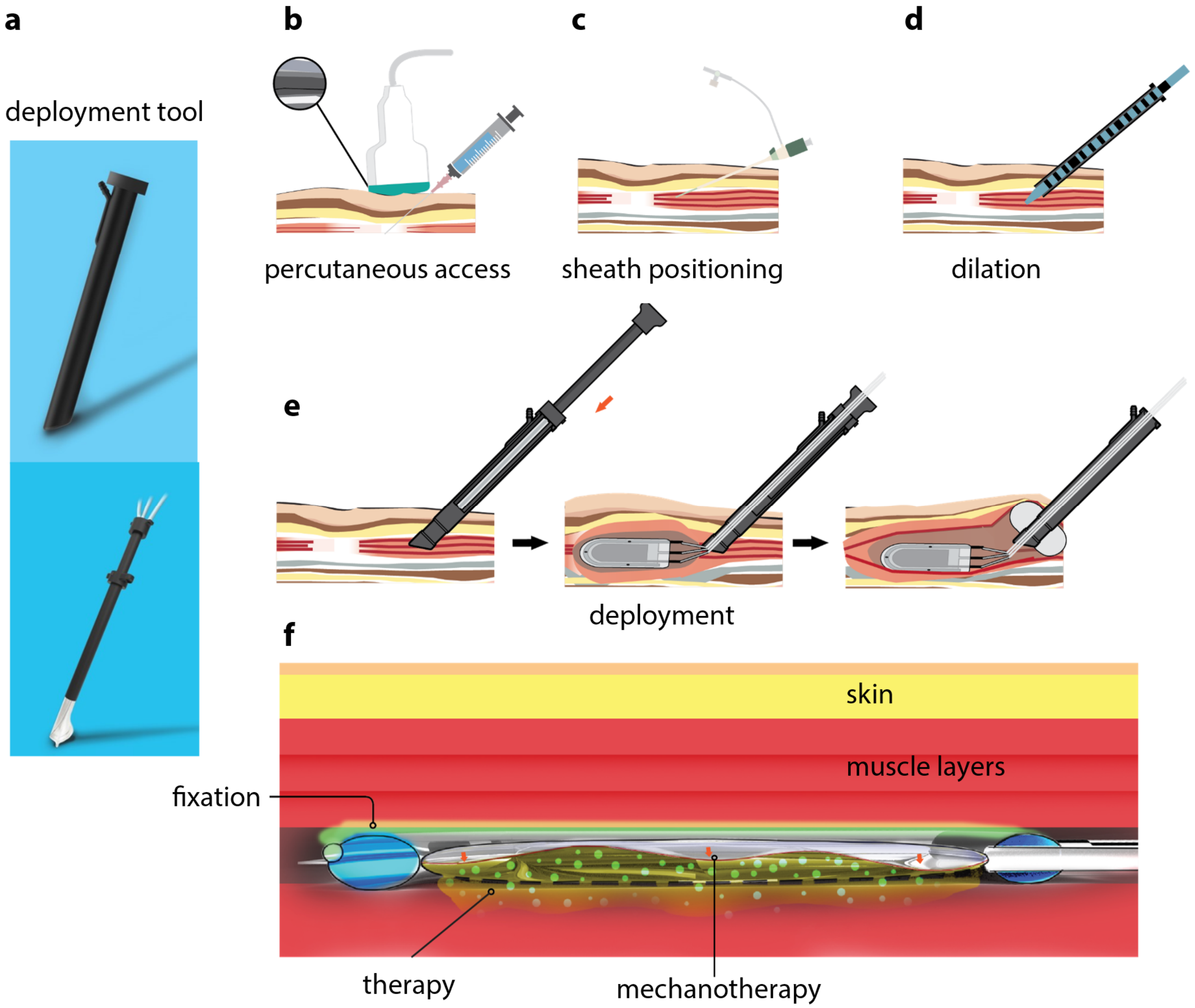

Supplementary Fig. 9 | Schematic for minimally invasive surgical implantation of human scale STAR device. a, CAD model of deployment tool. Top: Inflation sheath with side port for hand or CO_2 insufflation bulb. Bottom: Deployment plunger showing device in sheath and partially deployed with plunger. **b**, Ultrasound guidance is used to obtain needle access to the desired tissue plane and hydrodissection is performed to generate a potential space. **c**, A Seldinger technique is used to exchange the needle over a wire, and fluoroscopy is used to verify wire position in the tissue place. **d**, A commercially available dilator set is used to expand the space. **e**, Expanded space accommodates positioning of the deployment sheath. The deployment sheath is capped, and gas is used to pressurize the potential space while the deployment channel is filled with radiopaque contrast to enable opening of the device under fluoroscopic visualization. **f**, To secure the device in position, the adhesive channel is infused with a bioadhesive to fix the device to underlying tissue. Mechanical or biological therapy can then be delivered by the STAR device as required.

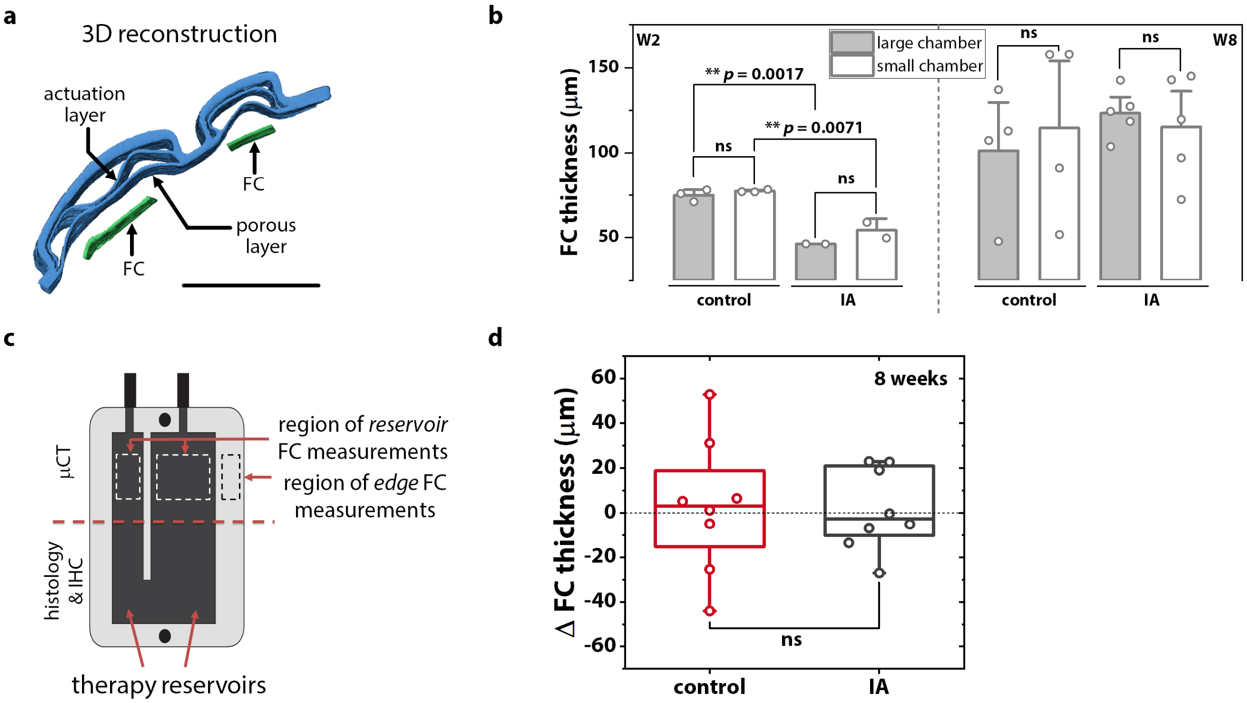

Supplementary Fig. 10 | Fibrous capsule thickness analysis using µCT. a, 3D reconstruction of STAR by µCT in the transverse plane, demonstrating small (3 mm) and large (6 mm) chambers of the device with fibrous encapsulation. Scale bar is 5 mm. **b,** Comparison of capsule thickness in large and small chambers at 2 weeks and 8 weeks. n = 2–5 with no technical replicates. Data are means ± standard error of mean. p-values are calculated from unpaired, one-tailed, two-sample t-tests. See Supplementary Note 1 for detailed statistical analyses. **c,** Schematic showing the STAR device with bi-chambered design. Dashed boxes denote areas of FC measurement underneath the 6 mm large and 3 mm small chambers (white) and at reservoir edge (black). **d,** Box and whiskers plot showing difference in mean FC Thickness adjacent to reservoir and edge regions of STAR (difference = reservoir – edge) measured at 8 weeks with n = 4 mice per group and one thickness measurement per chamber (i.e., 2 technical replicates). Box represents the inter-quartile range (25 – 75 percentile) and whiskers represent the minima and maxima. The middle horizontal line inside a box represents the median.

Supplementary Note 1 | Statistical Comparisons of Preclinical Data

Figure 3b.

Metric: AUC at Day 3 (% × min)

**group**	**time of measurement**	***n***	**mean**	**standard error of mean**
control	day 3	3	6805.99	261.78
IA	day 3	2	7626.03	704.16

<u>Statistical comparisons</u>

**compared groups**	***p*-value**	**is significant**
at day 3 (baseline): control vs IA	0.2193	no**

** Given no significant difference in insulin response between IA and control groups at day 3, we combined these data into one baseline group, which was used for all following comparisons. These five animals are plotted together in Figure 3c–f.

Figure 3c.

Metric: max. BG drop (%)

**group**	**time of measurement**	***n***	**mean**	**standard error of mean**
BL	day 3	5	-72.45	2.22
8W IA	week 8	5	-68.31	3.38
3W IA	week 8	5	-40.06	7.86
control	week 8	4	-20.95	4.31

<u>Statistical comparisons</u>

Total number of comparisons = m

- $m = 1$ for the first three rows below of within group comparisons,
- $m = 3$ for the last 3 rows of between group comparisons

Significance threshold at 95% confidence level = $0.05/m$

**compared groups/timepoints**	***p*-value**	**α/m**	**is significant**
within 8W IA group: BL vs 8 weeks	0.16757	0.05	no
within 3W IA group: BL vs 8 weeks	0.00207	0.05	yes
within control group: BL vs 8 weeks	0.00047	0.05	yes
at 8 weeks: 3W IA group vs control group	0.04450	0.017	no
at 8 weeks: 8W IA group vs 3W IA group	0.00541	0.017	yes
at 8 weeks: 8W IA group vs control group	0.00002	0.017	yes

Figure 3d.

Metric: Time to 30% blood glucose drop (min)

group	time of measurement	*n*	mean	standard error of mean
BL	day 3	5	10.89	1.80
IA	week 2	10	15.42	1.05
control	week 2	6	41.65	11.82
IA	week 3	10	21.32	1.23
control	week 3	6	48.95	10.98
8W IA	week 4	5	27.43	4.48
3W IA	week 4	5	38.06	9.01
control	week 4	6	73.55	14.85
8W IA	week 5	5	27.19	3.71
3W IA	week 5	5	32.69	4.46
control	week 5	5	77.34	18.69
8W IA	week 8	5	26.33	6.16
3W IA	week 8	5	65.76	22.24
control	week 8	4	>120	–

Figure 3f.

Metric: AUC at various timepoints (% × min)

group	time of measurement	*n*	mean	standard error of mean
IA	week 2	10	6492.05	319.91
control	week 2	6	4937.95	786.06
IA	week 3	10	5785.99	303.27
control	week 3	6	4054.31	364.21
8W IA	week 4	5	4615.08	533.96
3W IA	week 4	5	4177.47	452.79
control	week 4	6	2542.72	537.93
8W IA	week 5	5	4873.69	531.36
3W IA	week 5	5	3593.95	639.45
control	week 5	5	2155.62	578.70
8W IA	week 8	5	5802.30	522.71
3W IA	week 8	5	2892.71	796.39

control	week 8	4	971.82	424.36

Statistical comparisons

Total number of comparisons = m

- $m = 1$ for weeks 2 and 3 (single 2-group comparison at each timepoint).
- $m = 3$ for weeks 4–8 (3 comparisons between 3 groups at each timepoint).

Significance threshold at 95% confidence level = $0.05/m$

**compared groups**	***p*-value**	α/m	**is significant**
at week 2: control vs IA	0.0253	0.05	yes
at week 3: control vs IA	0.0015	0.05	yes
at week 4: control vs 8W IA	0.01205	0.017	yes
at week 4: control vs 3W IA	0.02487	0.017	no
at week 4: 3W IA vs 8W IA	0.27467	0.017	no
at week 5: control vs 8W IA	0.00429	0.017	yes
at week 5: control vs 3W IA	0.07965	0.017	no
at week 5: 3W IA vs 8W IA	0.06780	0.017	no
at week 8: control vs 8W IA	0.00012	0.017	yes
at week 8: control vs 3W IA	0.04499	0.017	no
at week 8: 3W IA vs 8W IA	0.00786	0.017	yes

Figure 4c.

Metric: Volume of Neutrophils (mm^3)

**group**	**time of measurement**	***n***	**mean**	**standard error of mean**
control	day 3	3	0.26133	0.02831
IA	day 3	3	0.23767	0.01894
control	day 5	3	0.34900	0.0589
IA	day 5	3	0.12567	0.0067

Statistical comparisons

**compared groups**	***p*-value**	**is significant**
at day 3: control vs IA	0.2627	no
at day 5: control vs IA	0.0098	yes

Figure 4e.

Metric: Volume of Myofibroblasts (mm^3)

group	time of measurement	*n*	mean	standard error of mean
control	week 2	3	0.03610	0.00229
IA	week 2	2	0.02185	0.00143

Statistical comparisons

compared groups	*p*-value	is significant
at week 2: control vs IA	0.0068	yes

Figure 4g.

Metric: Cells per capsular area (%)

group	time of measurement	*n*	mean	standard error of mean
control	day 3	3	0.7203	0.0168
IA	day 3	3	0.7957	0.1008
control	day 5	3	1.1907	0.2147
IA	day 5	3	1.0265	0.1587
control	week 2	3	1.7592	0.3925
IA	week 2	2	1.0426	0.0076

Statistical comparisons

compared groups	*p*-value	is significant
at day 3: control vs IA	0.7492	no
at day 5: control vs IA	0.2858	no
at week 2: control vs IA	0.1261	no

Figure 4i.

Metric: Fibrous capsule thickness (µm)

group	time of measurement	technical replicates*	*n*	mean	standard error of mean
control	day 3	2	6	53.8588	3.9472
IA	day 3	2	6	53.3199	4.0821
control	day 5	2	6	59.8096	7.1008
IA	day 5	2	6	50.9469	4.5207

control	week 2	2	6	76.2547	1.0824
IA	week 2	2	4	50.3887	2.9953
control	week 8	2	8	107.9733	15.1640
IA	week 8	2	10	119.3827	7.2912

* Technical replicates were two different measurements taken from fibrous capsule underlying each of the two chambers (large and small) for each device.

Statistical comparisons

compared groups/timepoints	*p*-value	is significant
at day 3: control vs IA	0.4631	no
at day 5: control vs IA	0.1713	no
at week 2: control vs IA	0.000006293	yes
at week 8: control vs IA	0.7602	no
within control group: day 3 vs week 2	0.00013617	yes
within IA group: day 3 vs week 2	0.6164	no

Figure 4k.

Metric: Collagen alignment by optical coherency (unitless)

group	time of measurement	technical replicates*	*n*	mean	standard error of mean
control	week 2	60	180	0.2912	0.0088
IA	week 2	60	120	0.3072	0.0118
control	week 8	60	240	0.3056	0.0071
IA	week 8	60	294^{+}	0.3893	0.0075

*Technical replicates were 6 ROIs per slice with 10 slices taken per sample.

$^{+}$ One slice comprised of 6 ROIs was of poor image quality and therefore was excluded from this analysis.

Statistical comparisons

compared groups/timepoints	*p*-value	is significant
at week 2: control vs IA	0.2720	no
at week 8: control vs IA	8.234E-15	yes
within control group: week 2 vs week 8	0.2027	no
within IA group: week 2 vs week 8	7.582E-9	yes

Supplementary Figure 7a.

Metric: Numerical density of blood vessels per unit area (N/mm^2)

group	time of measurement	*n*	mean	standard error of mean
control	week 2	3	718.6643	23.0575
IA	week 2	2	594.1995	27.4345
control	week 8	4	475.1421	93.7431
IA	week 8	5	525.8135	45.2984

<u>Statistical comparisons</u>

compared groups	*p*-value	is significant
at week 2: control vs IA	0.041	yes
at week 8: control vs IA	0.6177	no

Supplementary Figure 7b.

Metric: Diffusion distance (µm)

group	time of measurement	*n*	mean	standard error of mean
control	week 2	3	14.8913	0.2346
IA	week 2	2	16.3804	0.3796
control	week 8	4	19.1932	2.0328
IA	week 8	5	17.5946	0.7657

<u>Statistical comparisons</u>

compared groups	*p*-value	is significant
at week 2: control vs IA	0.037	yes
at week 8: control vs IA	0.4472	no

Supplementary Figure 7c.

Metric: Relative integrated density of yellow & green immature collagen fibres (unitless)

group	time of measurement	*n*	mean	standard error of mean
control	week 2	3	0.1788	0.0197
IA	week 2	2	0.1631	0.0814
control	week 8	4	0.0160	0.0074
IA	week 8	5	0.0155	0.0114

Statistical comparisons

compared groups	*p*-value	is significant
at week 2: control vs IA	0.8265	no
at week 8: control vs IA	0.9739	no

Supplementary Figure 7d.

Metric: Relative integrated density of red & orange mature collagen fibres (unitless)

group	time of measurement	*n*	mean	standard error of mean
control	week 2	3	0.8211	0.0197
IA	week 2	2	0.8369	0.0814
control	week 8	4	0.9349	0.0453
IA	week 8	5	0.9793	0.0111

Statistical comparisons

compared groups	*p*-value	is significant
at week 2: control vs IA	0.8261	no
at week 8: control vs IA	0.3218	no

Supplementary Figure 7e.

Metric: Radiodensity of fibrous capsule (Hounsfield units)

group	time of measurement	technical replicates*	*n*	mean	standard error of mean
control	week 8	2	8	6423.65	578.78
IA	week 8	2	10	7130.87	285.65

* Technical replicates were two different measurements taken from fibrous capsule underlying each of the two chambers (large and small) for each device.

Statistical comparisons

compared groups	*p*-value	is significant
at week 8: control vs IA	0.2602	no

Supplementary Figure 10b.

Metric: Fibrous capsule thickness underlying large and small chambers (μm)

group	chamber	time of measurement	*n*	mean	standard error of mean

control	large	week 2	3	75.0661	2.0597
control	small	week 2	3	77.4433	0.4499
IA	large	week 2	2	46.3545	0.0275
IA	small	week 2	2	54.4230	4.619
control	large	week 8	4	101.2995	18.9527
control	small	week 8	4	114.6471	26.1571
IA	large	week 8	5	123.3375	6.3065
IA	small	week 8	5	115.4279	13.8433

Statistical comparisons

**compared groups/timepoints**	***p*-value**	**is significant**
at week 2 within control group: large vs small	0.3226	no
at week 2 within IA group: large vs small	0.2224	no
at week 8 within control group: large vs small	0.6938	no
at week 8 within IA group: large vs small	0.6172	no
at week 2 within large chamber: control group vs IA group	0.0017	yes
at week 2 within small chamber: control group vs IA group	0.0071	yes

Supplementary Figure 10d.

Metric: Difference in FC thickness between reservoir and edge (μm)

**group**	**time of measurement**	**technical replicates***	***n***	**mean**	**standard error of mean**
control	week 8	2	8	2.8059	10.6681
IA	week 8	2	8	1.4898	6.5132

* Technical replicates were two different measurements taken from fibrous capsule underlying each of the two chambers (large and small) for each device.

Statistical comparisons

**compared groups**	***p*-value**	**is significant**
at week 8: control vs IA	0.9176	no
