# Supplementary material for: Dynamic actuation enhances transport and extends therapeutic lifespan in an implantable drug delivery platform
Source: Nat Commun. 2022 Aug 3;13:4496. doi: 10.1038/s41467-022-32147-w (PMC9349266; doi:10.1038/s41467-022-32147-w)
Supplement: Supplementary file 3 — Reporting Summary [file 41467_2022_32147_MOESM3_ESM.pdf]

nature portfolio

Corresponding author(s): Ellen T. Roche; Eimear B. Dolan

NCOMMS-21-31256C

Last updated by author(s): Jul 1, 2022

Reporting Summary

Nature Portfolio wishes to improve the reproducibility of the work that we publish. This form provides structure for consistency and transparency in reporting. For further information on Nature Portfolio policies, see our Editorial Policies and the Editorial Policy Checklist.

Statistics

For all statistical analyses, confirm that the following items are present in the figure legend, table legend, main text, or Methods section.

n/a	Confirmed	
☐	☒	The exact sample size (n) for each experimental group/condition, given as a discrete number and unit of measurement
☐	☒	A statement on whether measurements were taken from distinct samples or whether the same sample was measured repeatedly
☐	☒	The statistical test(s) used AND whether they are one- or two-sided<br>*Only common tests should be described solely by name; describe more complex techniques in the Methods section.*
☒	☐	A description of all covariates tested
☐	☒	A description of any assumptions or corrections, such as tests of normality and adjustment for multiple comparisons
☐	☒	A full description of the statistical parameters including central tendency (e.g. means) or other basic estimates (e.g. regression coefficient) AND variation (e.g. standard deviation) or associated estimates of uncertainty (e.g. confidence intervals)
☐	☒	For null hypothesis testing, the test statistic (e.g. F, t, r) with confidence intervals, effect sizes, degrees of freedom and P value noted<br>*Give P values as exact values whenever suitable.*
☒	☐	For Bayesian analysis, information on the choice of priors and Markov chain Monte Carlo settings
☒	☐	For hierarchical and complex designs, identification of the appropriate level for tests and full reporting of outcomes
☐	☒	Estimates of effect sizes (e.g. Cohen's d, Pearson's r), indicating how they were calculated

Our web collection on statistics for biologists contains articles on many of the points above.

Software and code

Policy information about availability of computer code

Data collection	Blood glucose measurements were collected using a commercial Bayer Contour Next Blood Glucose Monitoring System.
Data analysis	-Fluid-structure interaction simulations and finite element models of tissue strain were performed in Abaqus/Explicit 2018. Mass transport simulations were conducted using COMSOL Multiphysics 5.6 software.<br>-Diffusion area in fluorescent IVIS images were computed using the region of interest (ROI) function in the Living Image 4.5.4 Software (PerkinElmer)<br>-Photoacoustic ultrasound images were rendered and analyzed in VevoLAB 3.2.0 (Fujifilm).<br>-Cell number was assessed using Ocular 2.0 Imaging software on an Olympus microscope and immunofluoresence-stained slides were observed using a spinning disc inverted confocal microscope combined with Andor iQ 2.3 software.<br>-Fraction of aSMA+ cells within the fibrous capsule was estimated by an unbiased stereological counting technique using ImageJ (Fiji version 2.0.0) software<br>-Collagen fiber orientation analysis was performed using the OrientationJ 2.0.5 plugin (Biomedical Imaging Group, EPFL, Switzerland) for ImageJ (version 1.53k)<br>-Fibrous capsule thickness was quantified using Materialise MIMICS Research 18.0.0.525 and Materialise 3-matic Research 10.0.0.212 software<br>-All statistical analyses were performed in OriginPro 2018b (OriginLab Corp.) and the same software was used to generate all plots

For manuscripts utilizing custom algorithms or software that are central to the research but not yet described in published literature, software must be made available to editors and reviewers. We strongly encourage code deposition in a community repository (e.g. GitHub). See the Nature Portfolio guidelines for submitting code & software for further information.

Data

Policy information about availability of data

All manuscripts must include a data availability statement. This statement should provide the following information, where applicable:

- Accession codes, unique identifiers, or web links for publicly available datasets
- A description of any restrictions on data availability
- For clinical datasets or third party data, please ensure that the statement adheres to our policy

All data supporting the findings of this study are available within the article and the Supplementary Information.

Field-specific reporting

Please select the one below that is the best fit for your research. If you are not sure, read the appropriate sections before making your selection.

☒ Life sciences ☐ Behavioural & social sciences ☐ Ecological, evolutionary & environmental sciences

For a reference copy of the document with all sections, see nature.com/documents/nr-reporting-summary-flat.pdf

Life sciences study design

All studies must disclose on these points even when the disclosure is negative.

Sample size

Based on sample size of n = 3-7 used in published literature describing similar evaluations[1-3], we chose a starting sample size of n=5-6 animals per experimental group for our long-term implantation study to account for potential device failure or loss of mice at intermediate time-points and to ensure adequate sample of >3 mice per group for capsular and cellular analyses at the 8 week time point.

For our shorter-term mechanistic study investigating the acute inflammatory response and capsular thickness at days 3, 5, and 14 days post device implantation, we chose a sample size of 3 mice per experimental group based on the dramatic differences observed in our previous work applying the same regimen of mechanical actuation[4].

1.Carper, D., Coué, M., Laurens, C., Langin, D. & Moro, C. Reappraisal of the optimal fasting time for insulin tolerance tests in mice. Molecular Metabolism vol. 42 101058 (2020).
2. Walz, H. A. et al. Early and rapid development of insulin resistance, islet dysfunction and glucose intolerance after high-fat feeding in mice overexpressing phosphodiesterase 3B. Journal of Endocrinology vol. 189 629–641 (2006).
3. Siersbæk, M. S. et al. C57BL/6J substrain differences in response to high-fat diet intervention. Scientific Reports vol. 10 (2020).
4. Dolan, E. B. et al. An actuatable soft reservoir modulates host foreign body response. Science Robotics vol. 4 (2019).

Data exclusions

No data were excluded for insulin studies, histologic, immunofluorescence, microCT or SEM analyses.

Replication

In vivo studies were reliably reproduced both within and across groups, without periprocedural complications. The experiments were performed independently twice across the initial submission and revision.

Randomization

Experimental groups were randomly allocated post-surgery.

Blinding

-Animal experiments were not blinded as the same investigators performing insulin transport tests (ITTs) were performing actuations twice a day on the same animals. Animals were identified by tail markings and housed separately, but had to be identified by experimental group to ensure that actuation was performed on the correct mice.

-All histological analysis was conducted by blinded assessment.

-All fibrous capsule thickness measurements were repeated by two blinded operators.

-SEM and collagen fibre orientation analyses were not blinded, as these were post-hoc analyses that were performed after initial hypotheses around fibrous capsule thickness and vascularity were disproven.

Reporting for specific materials, systems and methods

We require information from authors about some types of materials, experimental systems and methods used in many studies. Here, indicate whether each material, system or method listed is relevant to your study. If you are not sure if a list item applies to your research, read the appropriate section before selecting a response.

Materials & experimental systems

n/a	Involved in the study
☐	☒ Antibodies
☒	☐ Eukaryotic cell lines
☒	☐ Palaeontology and archaeology
☐	☒ Animals and other organisms
☐	☒ Human research participants
☒	☐ Clinical data
☒	☐ Dual use research of concern

Methods

n/a	Involved in the study
☒	☐ ChIP-seq
☒	☐ Flow cytometry
☒	☐ MRI-based neuroimaging

Antibodies

Antibodies used

Primary antibodies:
Recombinant anti-CD31 antibody [EPR17259] (ab182981, Abcam); Anti-alpha smooth muscle Actin antibody [1A4] (ab7817, Abcam); Ly-6G antibody [1A8] (127602, Biolegend)

Secondary antibodies:
Alexa Fluor 594 goat anti-mouse IgG (A-11020, Thermo Fisher Scientific); Alexa Fluor 594 goat anti-rabbit IgG (A-11072, Thermo Fisher Scientific); Alexa Fluor 488 goat anti-mouse IgG (A-11001, Thermo Fisher Scientific); Alexa Fluor 488 goat anti-rat IgG (A-11006 Thermo Fisher Scientific).

Validation

All antibodies are commercially available, and have been tested by the manufacturer.

Rabbit anti-CD31 antibody [EPR17259] (ab182981, Abcam): This monoclonal antibody recognizes CD31. Manufacturer-validated to react with Mouse, Rat, Human CD31 (https://www.abcam.com/cd31-antibody-epr17259-ab182981.html).

Anti-alpha smooth muscle Actin antibody [1A4] (ab7817, Abcam): This monoclonal antibody recognizes aSMA. Manufacturer-validated to react with Mouse, Rat, Rabbit, Human, Pig aSMA (https://www.abcam.com/alpha-smooth-muscle-actin-antibody-1a4-ab7817.html).

Anti-Ly-6G antibody [1A8] (127602, Biolegend): This monoclonal antibody recognizes Ly-6G. Manufacturer validated to bind mouse Ly-6G (https://www.biolegend.com/en-gb/products/purified-anti-mouse-ly-6g-antibody-4767).

Animals and other organisms

Policy information about studies involving animals; ARRIVE guidelines recommended for reporting animal research

Laboratory animals

Male C57BL/6 mice (25-30g weight, 12 weeks old) were purchased from Charles River Laboratories.
Female Sprague Dawley rats (250–300g weight, 12 weeks old) were purchased from Charles River Laboratories.

Rodents were housed in a facility with 12 hour on/off light cycle, at 68-72°F with a humidity ranging between 30-70%. Animals were singly housed for the duration of the study, with standard bedding and food.

Wild animals

This study did not involve wild animals.

Field-collected samples

This study did not involve field-collected samples.

Ethics oversight

Animal procedures were reviewed and approved according to ethical regulations by the Institutional Animal Care and Use Committee at Massachusetts Institute of Technology.

Note that full information on the approval of the study protocol must also be provided in the manuscript.

Human research participants

Policy information about studies involving human research participants

Population characteristics

All cadavers used were adult males. Additional demographic information is not available for the cadavers involved, as they are de-identified when bequeathed to the Medical School.

Recruitment

All cadavers were bequeathed to the Medical School of National University of Ireland Galway for further advancement of medical knowledge. This is covered by legislation governing the practice of Anatomy in the Republic of Ireland (Medical Practitioners Act 2007).

Ethics oversight	The protocol was approved by the NUI Galway Research Ethics Committee.

Note that full information on the approval of the study protocol must also be provided in the manuscript.
