# Supplementary material for: Dynamic actuation enhances transport and extends therapeutic lifespan in an implantable drug delivery platform
Source: Nat Commun. 2022 Aug 3;13:4496. doi: 10.1038/s41467-022-32147-w (PMC9349266; doi:10.1038/s41467-022-32147-w)
Supplement: Supplementary file 4 — Description of Additional Supplementary Files [file 41467_2022_32147_MOESM4_ESM.docx]

**Description of Additional Supplementary Files**

**Supplementary Movie 1:** Intermittent actuation regimen using STAR in vitro.

**Supplementary Movie 2:** Actuationmediated rapid release using STAR in vitro.

**Supplementary Movie 3:** Actuationmediated rapid release using STAR in vivo recorded by photoacoustic imaging.
